# Persistent and Recurrent Bacterial Bronchitis—A Paradigm Shift in Our Understanding of Chronic Respiratory Disease

**DOI:** 10.3389/fped.2017.00019

**Published:** 2017-02-15

**Authors:** Alya Ishak, Mark L. Everard

**Affiliations:** ^1^Department of Respiratory Medicine, Princess Margaret Hospital, Subiaco, WA, Australia; ^2^University of Western Australia, Crawley, WA, Australia

**Keywords:** protracted bacterial bronchitis, recurrent bronchitis, bronchiectasis, chronic cough, endobronchial infection

## Abstract

The recent recognition that the conducting airways are not “sterile” and that they have their own dynamic microbiome, together with the rapid advances in our understanding of microbial biofilms and their roles in the causation of respiratory diseases (such as chronic bronchitis, sinusitis, and chronic otitis media), permit us to update the “vicious circle” hypothesis of the causation of bronchiectasis. This proposes that chronic inflammation driven by persistent bacterial bronchitis (PBB) causes damage to both the epithelium, resulting in impaired mucociliary clearance, and to the airway wall, which eventually manifests as bronchiectasis. The link between a “chronic bronchitis” and a persistence of bacterial pathogens, such as non-typable *Haemophilus influenzae*, was first made more than 100 years ago, and its probable role in the causation of bronchiectasis was proposed soon afterward. The recognition that the “usual suspects” are adept at forming biofilms and hence are able to persist and dominate the normal dynamically changing “healthy microbiome” of the conducting airways provides an explanation for the chronic colonization of the bronchi and for the associated chronic neutrophil-dominated inflammation characteristic of a PBB. Understanding the complex interaction between the host and the microbial communities of the conducting airways in health and disease will be a key component in optimizing pulmonary health in the future.

## Introduction

Our understanding of the role of bacteria in pulmonary disease is undergoing a transformation that will hopefully, in time, take pulmonary medicine into a new era of clarity regarding pulmonary health and disease. The idea that a persistent bacterial “infection” of the lower airways could be a cause of disease, characterized by a chronic cough, is not new. Excellent clinical descriptions of the disease that many now refer to as protracted or persistent bacterial bronchitis (PBB) long predated the recognition that bacteria might drive the disease. Physicians generally used terms such as chronic catarrh or chronic bronchitis (1–7), although numerous other names have also been used to describe the same condition including “pre-bronchiectasis,” chronic endobronchial infection, chronic suppurative lung disease, and indeed bronchiectasis, when used as a descriptor of a disease rather than a pathological appearance (8). Almost universally, it was noted that this was a common condition in childhood, often commencing in infancy or the very early years of life, usually after an acute bronchitis failed to resolve. It was particularly common in the poorer sections of society. During the early years of the twentieth century, it was established that a number of bacterial organisms were commonly identified in the sputum of individuals with chronic pulmonary diseases, including bronchiectasis and chronic bronchitis (9–11). The advent of antibiotics was associated with a rapid reduction in both the number of children admitted to hospital with “bronchiectasis” (12) and the number of children being diagnosed with “bronchiectasis” due to causes other than cystic fibrosis (CF) and immunodeficiency, such that many considered it an orphan disease from a by-gone era, at least in developed countries (13). Similarly by the 1980s, chronic bronchitis in childhood was felt to be a very uncommon condition (14, 15). This, together with a focus on asthma as the dominant cause of chronic respiratory symptoms in childhood, led many [though not all (16)] to dismiss the concept of a chronic endobronchial infection as a cause of chronic morbidity. This position was reinforced by their belief that the lungs were “sterile” and that bacteria caused acute illnesses that fully resolved, despite both propositions having been shown to be false decades before.

Over recent years, there has been a revival of interest in the role of persistent bacterial “infection” of the lower airways, as a cause of chronic cough and associated morbidity. In part, this would appear to be due to a genuine increase in prevalence, though the lack of a simple diagnostic test makes it impossible to substantiate this clinical suspicion. In parallel, our concepts of the behavior of bacteria in a biological niche, such as the conducting airways, have changed fundamentally with the belated recognition that the lungs are not a uniquely sterile environment (17, 18) and a recognition of the importance of bacterial biofilms in persistence of bacterial communities (19, 20).

## A “Vicious Circle” in the Context of a Respiratory Microbiome

The most plausible paradigm based on current knowledge is that a “chronic” bacterial bronchitis develops when one or more “pathogens” are able to establish themselves in the form of biofilms within the conducting airways (21). In this way, they come to dominate a niche, which under normal conditions appears to contain a dynamic microbial community that is constantly being cleared and replenished, predominantly by micro-aspiration but also through inhalation (22, 23). The persistence and consequent dominance of the local microbial ecology of the monospecies or mixed populations within the biofilms drive a chronic inflammatory state, which is of benefit to organisms such as non-typable *Haemophilus influenzae* (NTHi). NTHi utilizes products such as the DNA within neutrophil nets, for both nutritional and matrix building purposes, and will also benefit from the release of other nutrients into the local environment driven by the inflammation (20, 24). Moreover, the stimulated host response helps the NTHi compete with other potential pathogens such as *Streptococcus pneumoniae* (25) and with the normal “microbiota,” such that the diversity within the airways falls. Viral infections appear to not only enable the initiation of surface attachment and initiation of biofilms but also be the trigger for “exacerbations” characterized by the release of planktonic organisms, which will generate an enhanced inflammatory response (26, 27). This makes biological sense in that the viral infection will provide an enhanced opportunity to be transferred to another potential host and permit further expansion within the current host resulting in an “exacerbation” of symptoms beyond that attributable to the natural course of the viral illness.

Unraveling the dynamics of microbial and host interactions through the life course is likely to be a dominant theme in the coming decades as we work toward a focus on wellness and disease prevention. More importantly, this understanding will help develop a rational approach to promoting respiratory health though prevention and non-antibiotic-dependent strategies to therapy.

## Is “PBB” A Distinct Disease?

A persistent endobronchial infection (PBB) is a well-recognized feature and indeed is the defining clinical feature of the progressive pulmonary disease observed in patients with CF, primary ciliary dyskinesia (PCD), and agammaglobulinemia.

It has been suggested that the term PBB is used to describe a persistent endobronchial infection, in which a major underlying inherited risk factor has not been identified.^1^ This is somewhat of an arbitrary distinction but does have a degree of clinical validity in that it carries an expectation that the condition can be cured and the adverse long-term consequences prevented. As such, it would imply that in most cases, the development of bronchiectasis is entirely preventable and, if it does develop, is likely to be attributable to failure of clinicians to intervene appropriately.

The use of the term “PBB,” in this sense, still implies an ongoing inflammatory process driven by the persistence of “pathogenic” bacteria, for which there would be one or more factors contributing to its inception. As noted above, the lungs are not “sterile” as previously thought. What characterizes the microbial communities in those with an ongoing endobronchial infection is dominance of the microbial community by one or more “pathogens,” resulting in a significant reduction in the bacterial diversity observed within the microbial ecology of the conducting airways. If PBB implies the development of this process in the absence of the above inherited factors, it is still incumbent on the clinician to not only treat the infection but to also consider whether any risk factors are ongoing and require addressing.

While most cases appear to be “post-infectious,” anything that impairs mucociliary clearance is a potential risk factor. This includes damage to the ciliated epithelium through recurrent aspiration, poorly controlled asthma, or inhaled toxins such as tobacco smoke. A second group is represented by structural issues such as tracheo- and/or bronchomalacia, as well as bronchial narrowing, the latter which includes narrowing due to mucosal swelling secondary to large left-to-right shunts (such as may occur with a ventricular septal defect) and impingement from dilated or abnormally sited blood vessels. Relatively minor immune impairments such as low IgG subclasses or low MBL levels also appear to be a risk factor, while acquired defects, such as that which develop during treatment for oncological conditions, are another relatively common cause. None of these inevitably lead to the development of PBB, in contrast to the major inherited conditions outlined above.

In the past, the condition was attributed to failure of an acute bronchitis or bronchopneumonia to resolve. Pertussis and measles infections were felt to be particularly likely to facilitate the onset of a chronic bronchitis (and in time progression to bronchiectasis) but accounted for a minority of cases. Both result in significant damage to cilia, as do significant viral infections, and it is likely that currently most cases of PBB develop following a viral lower respiratory tract infection (LRTI). NTHi, for example, is unable to adhere to a healthy differentiated epithelium and hence appears to be opportunistic in the face of damage to the ciliated epithelium that occurs during these infections (28). Significant symptomatic viral LRTIs are most common in the first few years of life, which in part provides an explanation for the predilection of the condition to commence in early life, though structural issues and relative immune defects will also play a role. The impact of improved nutrition and living conditions that occurred post the second world war, together with vaccination and the widespread use of antibiotics to “prevent secondary infection” (as was advocated by many in the early antibiotic era), led to the apparent disappearance of “chronic bronchitis of childhood” and bronchiectasis in the subsequent few decades.

In light of this, one can question whether the use of PBB as a discrete term can really be justified. A more pragmatic response may be to accept that a persistent endobronchial infection may be due to one of the major risk factors, which almost inevitably lead to the development of persistent or recurrent bacterial bronchitis (such as CF, PCD, and agammaglobulinemia) or one of the many conditions that predispose but do not inevitably lead to development of the condition (such as aspiration, chemotherapy, bronchomalacia, poorly controlled asthma, large left-to-right cardiac shunt, pertussis, mild impairments of innate and adaptive immunity, heavy exposure to tobacco smoke, persistent foreign body, etc.).

Throughout the past two centuries, physicians have emphasized the role of poverty and the related overcrowding, poor nutrition and “poor air” in the development of chronic bronchitis in children—these factors still appear to be relevant at least in Indigenous and less developed countries (29). However, the condition is far from restricted to particular social groups and in many children, no significant risk factors are identified (even after investigation), and hence, these are likely to be post-infectious, most commonly after a viral LRTI.

## PBB and Its Relationship to Bronchiectasis

Bronchiectasis is not a disease but a radiological or pathological appearance. Lannec’s landmark publication of 1818 (1) provides the first description of bronchiectasis in a post-mortem sample. He observed that “*the chronic catarrh is sometimes accompanied with a general or partial dilatation of the bronchia*” and “*the dilatation of the bronchia is only met with in cases of chronic mucous catarrh*.” Thus, he correctly and robustly established the link between chronic bronchitis and the development of “bronchiectasis.” His observations long predated the identification of bacteria, and he speculated that the changes were due to pressure effects of the locally produced mucus.

The idea of a vicious cycle of inflammation, driven by infection, leading to damage, and thereby predisposing to ongoing chronic inflammation, has long formed the basis for our understanding of how bronchiectasis develops in most individuals with the condition (30–34). Of course, there are other mechanisms including the acute onset associated with post-infectious obliterative bronchiolitis in early childhood, severe bacterial pneumonic infections such as those due to PVL toxin-producing *Staphylococcus aureus*, and inflammatory conditions such as inflammatory bowel disease. The concept of aggressive treatment of a chronic bronchitis to prevent progression to bronchiectasis is also long standing (31, 35). In all publications from Lannec onward, there is clear guidance that complete resolution of an acute bronchitis/catarrh was necessary in order to prevent the establishment of a chronic bronchitis. In 1947, Patterson and Moncrieff noted that when discussing the prevention of bronchiectasis, “*it is important to realise a cough persisting in a child longer than a few weeks requires full investigation and early treatment*.” (31)

Many patients will have radiological bronchiectasis and feel well between exacerbations. From the earliest reports, physicians have observed that many patients with bronchiectasis were well much of the time but prone to “bronchitis” in the winter. Furthermore, the majority of morbidity associated with “bronchiectasis” appears to be directly attributable to the associated persistent or recurrent endobronchial infection, which is the cause of the disease (35–37). Conversely, the belief that all is well if the CT scan does not demonstrate bronchiectasis is one of the many fallacies that has bedeviled this field over the past 20 years and led to missed opportunities to prevent the development of the structural damage that eventually becomes apparent on high-resolution CT. The documented progression from “pre-bronchiectasis” to bronchiectasis based on sequential bronchograms was described more than half a century ago (38). This “progression” was not marked by any significant change in symptoms, and the author concluded that one could not distinguish pre-bronchiectasis and bronchiectasis on the basis of symptoms. It was also noted that clinical cure occurred in individuals, despite persistence of bronchiectatic changes, though regression and/or complete resolution of “bronchiectasis” was also noted, all of which reinforce the argument that bronchiectasis is in itself not a “disease” (39, 40). Some have suggested that persistence or recurrence of chronic cough is a marker for having developed bronchiectasis. However, the study did not have data at presentation but only in a selected minority when symptoms persisted; thus, it is impossible to interpret these data given the potential for changes to resolve and for the changes to have been present long before referral. Clinical objective evidence of bronchiectasis is useful when parents are struggling to take the diagnosis seriously, but the presence or absence of bronchiectasis has not been shown in children to clearly predict the amount of morbidity likely to be experienced in the short to medium term.

It is often forgotten that much of the pulmonary function impairment attributable to “bronchiectasis” is due to the obliteration of small airways distal to the bronchiectatic areas (41, 42). In large part, this is because the loss of distal branches is not evident when viewing the CT scans, in contrast to the obvious more central dilatation. The process is likely to be very similar with inflammation destroying tissues such as smooth muscle, elastic tissue, and cartilage in the walls of the larger airways, while the same inflammation in smaller airways results in obliteration of the lumen, due to the proximity of the opposite bronchial wall.

Bronchial wall thickening is commonly observed on CT scans prior to the development of overt bronchiectasis and is thought to be due to inflammation, though it is not yet possible to exclude the apparent thickening being due to increased luminal mucus. Such changes are commonly associated with peripheral gas trapping, particularly evident on expiratory films. The same appearance can be seen in poorly controlled asthmatics.

It has been known for decades that aggressive treatment can result in resolution of radiologically proven “bronchiectasis” changes, at least in those whose bronchiectasis is not too severe and in the absence of major underlying problems such as CF (16, 40, 43, 44). Hence, the view that bronchiectasis is “irreversible,” which is largely derived from adult clinics, is incorrect. The radiologically confirmed “bronchiectasis” can resolve with appropriate management and pre-dates the advent of antibiotics at a time when therapy was driven by airway clearance/physiotherapy, breathing exercises, nutrition, and fresh air.

## Prevalence

There are no prevalence data for this condition, which is a major problem when trying to assess its societal importance and justify research in this area. Using bronchiectasis as a marker of prevalence, given the probable role of a persistent endobronchial infection in the causation of so many cases of bronchiectasis, it would appear to be more common in third world and Indigenous populations (13, 45–47). A 1997 review of studies reporting the prevalence of cough among primary school children undertaken in the decades after introduction of antibiotics (48) included a 1996 study from Poland that reported prevalence of chronic cough of 31.9%, a 1996 study from the USA reporting 27.4% with a “persistent” cough, a 1995 study from the Netherlands reporting chronic cough in 12.6% of children, a study from Israel reporting chronic cough with sputum in 8.1%, a 1992 study from Italy giving a prevalence of cough and phlegm in 5.5%, and a Canadian study reporting chronic cough with phlegm in 5.3%. As noted above bronchiectasis, and by inference PBB, is common in South East Asia and Indigenous populations. There is little or no information regarding non-HIV- or TB-related bronchiectasis in Africa, causing some to suggest that it is a rare cause of morbidity on the continent. An alternative explanation is that the ascertainment is low. A recent study from Nigeria suggested that 16.6% of those aged 7–14 years have a chronic productive cough (49).

These studies, as with most asthma epidemiological studies, did not generally include detailed clinical assessment, so it is impossible to determine how many of the symptomatic children had an ongoing endobronchial bacterial infection. It appears the vast majority with a persistent dry cough will become symptom free without treatment over a number of months, and among these conditions, infection (such as pertussis) is a significant contributor (50). Hence, it is likely that the figures for those studies reporting chronic cough with phlegm may provide a better guide to the prevalence of PBB. However, a proportion of those with a chronic productive cough is likely to have poorly controlled or untreated asthma. A study by Carter et al. (51) found that 7.2% of 2,397 Canadians aged 11–15 years old met the criteria for a chronic bronchitis (similar to many studies reported in the review above). Of these, 47% appeared to have asthma on the basis of a self-reported wheeze assisted with video examples. This does not exclude subjects having asthma and a co-morbidity such as PBB, though it seems unlikely that this would account for all of them. Even excluding those who appeared to have asthma, 3.8% of the population had non-asthma related chronic bronchitis, which makes it a significant cause of morbidity.

A well-designed community-based study is urgently required to assess current levels of symptoms and to determine with greater accuracy the prevalence and natural history of conditions, such as PBB, in childhood.

## Information from Studies Conducted in Secondary Care

A number of studies published in the past decade involving children referred to secondary care with respiratory symptoms, such as chronic cough, difficult asthma, or recurrent chest infections, identified bacterial bronchitis to be perhaps the commonest diagnosis in those with a persistent “wet” cough (8, 51–59). A study involving 190 young children in whom the diagnosis was made after referral to secondary care (60) found that 80% had made 5 or more visits to the doctor in the previous 12 months and 53% had made 10 or more visits prior to referral, supporting the suggestion that the condition is under-recognized in primary care. This is perhaps not surprising, given the high rates of self-resolving acute “viral” respiratory infections in the community, which can be linked to the drive to reduce inappropriate antibiotic prescribing for self-limiting infections. Several large studies have indicated that at most, only a very small proportion of acute respiratory illness fail to resolve by 28 days, and of these, many will subsequently resolve (50, 61). All of this together with “opinion leaders” encouraging primary care physicians to focus on asthma reinforce the impression that acute respiratory illnesses are common but self-limiting, and that if symptoms are ongoing, they are probably “just another virus” or asthma.

There are likely to be high levels of unnecessary morbidity among at risk groups in the community (such as those with severe cerebral palsy) that are attributable to unrecognized and hence untreated ongoing bacterial bronchitis. However, it is salutary to note that even among those referred to a secondary care service with a “chronic wet cough” of greater than 3 weeks duration, more than 20% resolve without any specific interventions (53). It is quite possible, but far from certain, that many of these may have had a PBB. A small randomized trial of antibiotics for the treatment of children with a clinical PBB (62) found that there was a statistically significant difference in clearance of cough at 2 weeks between those treated with antibiotics and those not on antibiotics. However, it is of note that only 48% of those treated with co-amoxiclav cleared their cough, while 16% of those on placebo became cough free. Clearly, recognition that such “spontaneous” resolution can occur is very important in the future design of any intervention study.

This ability for symptoms consistent with a chronic bronchitis to resolve “spontaneously” has been recognized for hundreds of years. A consistent report over time is that it is common for those with “chronic bronchitis” to become symptom free in the summer, and it is not uncommon to hear parents observe that their child’s cough resolves when on their summer holidays. Indeed, clinicians have been recommending that patients with a “chronic bronchitis” move to warm dry climates, should “conventional” therapy fail since at least the early nineteenth century.

## Resolution and Natural History

The elephant in the room in this field is: if “PBB” is a true entity that may sooner or later lead to the development of radiologically diagnosed bronchiectasis, why do most of the subjects appear to be pre-school children, with relatively few presenting at a later age? This would suggest that children who are symptomatic for extended periods of time are able to recover as the number of viral LRTI falls and their immunity develops, which is consistent with the observation above. Alternatively, that the symptoms generally become less troublesome with increasing age, again probably due to reduced exacerbations from viral infections and immunological changes, and increasing activity and possibly other undefined factors. There is certainly evidence for the latter in that follow-up of children with established bronchiectasis being treated in hospital in the 1950s found that symptoms and adverse impact on quality of life generally fell markedly through the second decades, and this improvement was maintained into the next decades (12). However, many patients now turning up in adult “bronchiectasis” clinics in middle age are probably experiencing deterioration of a condition, whose roots go back into childhood.

A further factor in the reduction of presentations in the primary school age and beyond is that the child and their family learn to live with it—they will report being fine despite significant symptoms because they are so much better than when younger. The lack of help following repeated presentations to doctors contributes to an acceptance that nothing can be done, while many parents simply tune out their child’s cough as it becomes part of their life. The study from Seattle (51) suggests that this may be a major factor, given that the prevalence of chronic bronchitis was 7.2% in 7- to 14-year olds, yet very few will have been subject to detailed clinical assessment.

## Diagnostic Challenges

The clinical features of the condition and the challenges in interpreting aspects of both the history and examination have been discussed elsewhere (8). At present, we do not have a simple non-invasive test that permits a robust diagnosis, and hence, it is inevitable that over- and under-diagnosis will be relatively common, as is still the case for asthma.

The so-called “definitive test” is a bronchoscopy and bronchoalveolar lavage (BAL), but interpretation is fraught with difficulty.

(a) Different laboratories report BAL differently using “quantitative” (e.g., 3 × 10^4^) or qualitative (e.g., “moderate growth”) methods to indicate the likely bacterial load. Such approaches, particularly the quantitative approach, were developed in the context of acute planktonic bacterial infections such as acute urinary tract infections or acute lobar pneumonia. Currently, there is no accepted approach to interpretation of culture data in the diagnosis of biofilm diseases (63). Given that a chronic endobronchial bacterial infection is likely to be a biofilm-dominated disease, this precludes the use of clear quantitative cut offs. A recent study which demonstrated the presence of biofilms in the lower airways of children with non-CF bronchiectasis also highlighted that the biofilms were often not present in the first lavage, presumably due to attachment of the biofilm to the epithelial surface (64).
(b) Conventional microbiology is in part a subjective area, and the author has long divided each BAL sample, knowing that commonly the same sample will produce a positive culture and a negative culture.
(c) Studies repeatedly report that approximately 25–30% of samples from children with a presumed PBB do not culture a “pathogen.” This may be due to the issues raised above. The impact of recent courses of antibiotics in suppressing bacterial metabolism and proliferation is another potential factor, and we commonly try to have patients free of antibiotics for 4–6 weeks before a bronchoscopy if possible. However, this is frequently not possible, and it is also common to culture “pathogens” even when apparently on antibiotics, perhaps attributable to increased release of planktonic organisms with exacerbations.
(d) Another potential explanation is that the “usual suspects” do not include the full range of biofilm-forming organisms that drive airway inflammation. The most likely culprits include members of the “normal oral flora” such as certain *Neisseria* species (65).
(e) As noted above, it is only recently that studies utilizing metagenomic sequencing have challenged the somewhat bizarre view that the lower airways are normally sterile. However, as with many aspects of respiratory medicine, we are coming full circle in that studies from the early twentieth century have consistently found bacteria such as *S. pneumoniae* in the lungs of “healthy” individuals. A number of studies appear to have confirmed that this and other “pathogens” can be isolated from BAL samples obtained from completely well children. If, as has been suggested, this is due to contamination of the bronchoscope as it traverses the upper airways, then it would call into question all culture results, as it will be impossible to know if the sample is contaminated or not. In a recent small (unpublished) study in which a bronchoscopy was undertaken *via* endotracheal tube inserted for an orthopedic procedure in a previously entirely well child, two of the four BAL samples cultured significant quantities of *S. pneumoniae* and/or NTHi.
(f) The use of qualitative neutrophil counts can help interpret the data, but again the range of “*% neutrophils”* in the differential cell count varies enormously. In some patients, the values can overlap with “normal” values presumably as a consequence of factors such as disease activity, recent antibiotics, and extent of disease across the extremely large surface area represented by the conduction airways. Conversely, a high percentage neutrophils among cells obtain by BAL may simply due to a viral infection. Infants with acute bronchiolitis, for example, have greatly elevated cell numbers in the BAL, and the vast majority of these are neutrophils with median of 76% neutrophils (66) being much higher than that seen in patients with “PBB.”

## Treatment—The Virtually Evidence-Free Zone

The most pragmatic guide to management is that if a child with a recurrent endobronchial bacterial infection is coughing, there is ongoing inflammation and at best the airways are not recovering. Conversely, if the child is not coughing, the airways are probably undergoing repair and healing. The goal of “cure” is to reach a point that the child consistently recovers from viral respiratory infections without the use of antibiotics at the same time as other children.

There is little in the literature to guide treatment, other than a general consensus that the cough will resolve in 10–14 days in the vast majority of subjects when treated with high-dose antibiotics such co-amoxiclav 40–50 mg/kg/day. Issues such as whether the course should be extended to provide opportunities for the airways to recover and if so, should this be 4, 6, or 26 weeks, have not been explored at all.

The initial course of treatment is aimed at both addressing the morbidity and being part of the diagnostic process, with a clear and unequivocal cessation of coughing being taken as confirmatory evidence of the putative diagnosis. However, as noted above, the response may have been a regression to the mean though if the cough does not reoccur, neither parent nor doctor worry overly. “Coughing less” is not sufficient and may simply be regression to the mean. Typically the child’s wellbeing improves in the first week, and the cough tends to resolve at around 10–14 days. Failure to respond may be because of incorrect putative diagnosis, poor adherence, or unidentified host or bacterial factors. In some who fail to respond to the above, the symptoms resolve with a change to therapy such as azithromycin, though why this should be when all the “usual” organisms are generally susceptible *in vitro* is unclear. There is also ongoing debate as to the role of a twice daily regime rather than a three times a day amoxicillin regime in some treatment failures. On occasions, much higher doses of amoxicillin appear to be required, necessitating the use of both co-amoxiclav and additional amoxicillin (up to 90 mg/kg/day of amoxicillin).

The use of macrolides does appear to be associated with increased incidence of antibiotic resistance (67).

Once the cough has resolved, it is assumed that the pathogenic organism has been eradicated, or at least the load of bacteria is greatly reduced, though it is quite likely that bacteria within biofilms further alter their metabolism, becoming virtually latent in order to deal with the antibiotic threat. A common experience is for symptoms to reoccur, which is not surprising, given the conceptual model above. Even in the absence of bronchiectasis, the conducting airways will be damaged from weeks, months, and years of neutrophilic inflammation. At the very least, the epithelial structure, including cilia, must recover and data from the literature suggest that recovery of ciliated epithelium after a brief acute viral infection (which is characterized by intense neutrophilic inflammation) can take weeks to months to fully recover (68).

Hence, it is perhaps not surprising that a bacterial bronchitis is frequently re-established, given that the major predisposing factor for a PBB is impaired mucocillary clearance, that the upper airways of children are often colonized by potential respiratory “pathogens,” and that oro-pharyngeal content is likely to be regularly aspirated. Given this conceptual model, many clinicians will continue the antibiotics for 6–8 weeks after resolution of the cough to permit repair of the airway structure, though some will stop at 2 weeks, while others report leaving patients on for 6 months or through the whole of the first winter following diagnosis. This model suggests that when the children are well and not coughing, they are likely to be repairing their airways. However, if they are coughing (other than the transient coughing with a viral infection), this signifies ongoing inflammation and the patients are failing to repair their airways but are also more likely to be contributing to further damage, which may progress to bronchiectasis evident on a CT scan or bronchogram. As with much in this area, further studies are required to confirm that this is indeed the most effective and pragmatic approach.

The idea that a long course of antibiotics might be required to treat respiratory patients other than those with CF, PCD, or non-CF bronchiectasis has been a challenge for many pulmonologists, though the use of “prophylactic” (i.e., long-term) antibiotics is widely practised by immunologists looking after children with “recurrent” infections, who do not have major immune defects.

There is no evidence base for any of these approaches, and research is urgently required. The challenge in designing studies is that the disease, as with asthma, has a spectrum of severity, from those in whom the condition persists for some weeks and months but resolves spontaneously to those in whom failure to make the diagnosis has resulted in established bronchiectasis. Anecdotally, in the absence of an identified major risk factor, the longer the symptoms have persisted, the longer it takes to clear the condition but this is far from reliable. This is presumably influenced by the extent of disease and risk factors such as relatively minor immunodeficiency in either the innate or humoral systems, overcrowding, frequencies of viral infections, and exposure to respiratory bacterial pathogens (such as in nurseries), as well as other factors that may play a role such as exposure to cigarette or other smoke. Adherence is of course likely to be a factor in some, though it is surprising how infrequent this appears to be the case for bacterial bronchitis compared to asthma. This may be because the improvement in quality of life for the individual and the family is such that parents are motivated and partly because, if explained clearly, the families are offered the potential of a cure rather than simply controlling a condition.

If longer courses are used, there must be a transition from requiring antibiotics to maintain health to a position where the condition is cured and the child can experience a viral respiratory infection without reoccurrence. This again varies from child to child with strategies being used including commencing a 2-week course at the onset of colds, then moving to waiting for 5–10 days to determine whether symptoms will resolve spontaneously, as individuals progress from persistent symptoms to cure.

The role of physiotherapy is also unclear. While the saying “*it is better out than in*” is undoubtedly as true in persistent bacterial infections as in the surgical drainage of an abscess, PBB will resolve in many with antibiotics alone. If the condition reoccurs, physiotherapy is likely (but not proven) to be helpful, as are strategies to try and minimize airway collapse in those with significant malacia, such as bubble positive expiratory pressure.

## Future

Recommending when to intervene with antibiotics in order to prevent the establishment of a bacterial bronchitis is challenging. It is important not to develop a position of treating all apparent viral infections in order to prevent the development of a chronic bronchitis (as was recommended in the early days of antibiotics), but it is equally clear that eradication is easier before the condition becomes chronic. With a median duration of approximately 6 days for most respiratory viruses being reported in all age groups and the vast majority of episodes resolving before 21 days, it may be reasonable to restrict antibiotics to those who have an ongoing wet cough at 3 weeks. Again, this is an entirely evidence-free zone and even this may mean the benefits to a few may be outweighed by the societal impact of antibiotics. Against this is the antibiotic use required to cure an individual of the condition, particularly if they ultimately progress to significant airway damage.

Developing rapid non-invasive testing to identify ongoing inflammation typical of a bacterial bronchitis or markers of persistence (for example, quorum-sensing molecules) using some of the newer “omics” technologies such as metabolomics, might ultimately permit the rational use of antibiotics for respiratory symptoms.

## Author Contributions

ME developed the concept for the review article and provided much of the background and references, especially for the historical aspects. AI contributed to the writing of the review and ensured flow of the manuscript.

## Conflict of Interest Statement

The authors declare that the research was conducted in the absence of any commercial or financial relationships that could be construed as a potential conflict of interest.

## Footnotes

^1^Kantar A, Chang AB, Shields MD, Marchant JM, Grimwood K, Grigg J, et al. ERS Task Force Document: protracted bacterial bronchitis in children TF-2015-03. *Eur Respir J* (Forthcoming).
